# Supplementary material for: Surprising phenotypic diversity of cancer-associated mutations of Gly 34 in the histone H3 tail
Source: eLife. 2021 Feb 1;10:e65369. doi: 10.7554/eLife.65369 (PMC7872514; doi:10.7554/eLife.65369)
Supplement: Supplementary file 2. [file elife-65369-supp2.docx]

**Supplementary File 2:** Peptides used for Antibody characterization and for mass spectrometry calibration.

| PP 33 | WT K36me2 | PATGGVK(me2)KPHRY-biotin |
| --- | --- | --- |
| PP 34 | WT K36me3 | PATGGVK(me3)KPHRY-biotin |
| PP 35 | WT K36 | PATGGVKKPHRY-biotin |
| PP36 | WT K36ac | PATGGVK(ac)KPHRY-biotin |
| PP 38 | G34R K36me2 | PATGRVK(me2)KPHRY-biotin |
| PP 39 | G34R K36me3 | PATGRVK(me3)KPHRY-biotin |
| PP 40 | G34R K36 | PATGRVKKPHRY-biotin |
| PP 41 | G34R K36ac | PATGRVK(ac)KPHRY-biotin |
| PP 42 | WT H3 cleavage | K(Ac)AAPATGGVK(Ac)K(Ac)PHR |
| PP 43 | G34V cleavage | K(Ac)AAPATGVVK(Ac)K(Ac)PHR |
| PP 44 | G34R cleavage | K(Ac)AAPATGR |
| PP 45 | G34R cleavage | VK(Ac)K(Ac)PHR |
| PP 46 | G34R cleavage | VK(prop)K(prop)PHR |
| PP 47 | G34V K36 | PATGVVKKPHRY-biotin |
| PP 49 | G34V K36me2 | PATGVVK(me2)KPHRY-biotin |
| PP 50 | G34V K36me3 | PATGVVK(me3)KPHRY-biotin |
| PP 51 | G34V K36ac | PATGVVK(ac)KPHRY-biotin |
| PP 52 | WT H3 cleavage | K(prop)AAPATGGVK(prop)K(prop)PHR |
| PP 53 | G34V cleavage | K(prop)AAPATGVVK(prop)K(prop)PHR |
| PP 54 | G34R cleavage | K(prop)AAPATGR |
| PP 55 | H3 cleavage | K(prop)AAPATGGVK(Ac)K(Ac)PHR |
| PP 63 | G34K K36me2 | PATGKVK(me2)KPHRY-biotin |
| PP 64 | G34K K36me3 | PATGKVK(me3)KPHRY-biotin |
| PP 65 | G34Q K36me2 | PATGQVK(me2)KPHRY-biotin |
| PP 66 | G34Q K36me3 | PATGQVK(me3)KPHRY-biotin |
| PP 67 | G34M K36me2 | PATGMVK(me2)KPHRY-biotin |
| PP 68 | G34M K36me3 | PATGMVK(me3)KPHRY-biotin |
| PP 69 | G34W K36me2 | PATGWVK(me2)KPHRY-biotin |
| PP 70 | G34W K36me3 | PATGWVK(me3)KPHRY-biotin |
| PP 71 | H3G34K cleavage | K(Ac)AAPATGKVK(Ac)K(Ac)PHR |
| PP 72 | H3G34K cleavage | K(prop)AAPATGKVK(prop)K(prop)PHR |
| PP 73 | H3G34K cleavage | K(Ac)AAPATGK(Ac)VK(Ac)K(Ac)PHR |
| PP 74 | H3G34K cleavage | K(prop)AAPATGK(prop)VK(prop)K(prop)PHR |
| PP 81 | H3G34K cleavage | K(Ac)AAPATGK(prop)VK(Ac)K(Ac)PHR |
| PP 82 | H3G34K cleavage | K(prop)AAPATGK(prop)VK(Ac)K(Ac)PHR |
| PP 83 | H3G34K cleavage | K(prop)AAPATGK(prop)VK(prop)K(Ac)PHR |
| PP 92 | G34K K36ac | PATGKVK(ac)KPHRY-biotin |
| PP 93 | G34K K36 | PATGKVKKPHRY-biotin |
