# Supplementary material for: Surprising phenotypic diversity of cancer-associated mutations of Gly 34 in the histone H3 tail
Source: eLife. 2021 Feb 1;10:e65369. doi: 10.7554/eLife.65369 (PMC7872514; doi:10.7554/eLife.65369)
Supplement: Supplementary file 3. [file elife-65369-supp3.docx]

**Supplementary File 3.** Detection parameters of unique tryptic peptides from *S.* *pombe* H3

| **WT H3** | **Precursor ion (m/z)** | | **Product ions (m/z)** | **Collision energy (eV)** | **Retention time (min)** | |
| --- | --- | --- | --- | --- | --- | --- |
| KpQLASKpAAR | | 542.827 | 772.468 | 19 | 4.35 |  |
|  | |  | 659.384 | 19 |  |  |
| KpQLASKaAAR | | 535.819 | 758.452 | 19 | 3.50 |  |
|  | |  | 645.368 | 19 |  |  |
| KaQLASKpAAR | | 535.821 | 772.468 | 19 | 3.50 |  |
|  | |  | 659.384 | 19 |  |  |
| KaQLASKaAAR | | 528.812 | 758.452 | 19 | 2.45 |  |
|  | |  | 645.368 | 19 |  |  |
| KpAAPATGGVKpKpPHR | | 529.311 | 1259.722 | 22 | 5.25 |  |
|  | |  | 933.563 | 22 |  |  |
|  | |  | 593.352 | 22 |  |  |
| KpAAPATGGVKpKaPHR | | 524.638 | 1245.706 | 22 | 4.85 |  |
|  | |  | 919.547 | 22 |  |  |
|  | |  | 579.336 | 22 |  |  |
| KpAAPATGGVKaKpPHR | | 524.640 | 1245.706 | 22 | 4.85 |  |
|  | |  | 919.547 | 22 |  |  |
|  | |  | 593.352 | 22 |  |  |
| KaAAPATGGVKpKpPHR | | 524.642 | 1259.722 | 22 | 4.85 |  |
|  | |  | 933.563 | 22 |  |  |
|  | |  | 593.352 | 22 |  |  |
| KpAAPATGGVKaKaPHR | | 519.967 | 1231.691 | 22 | 4.35 |  |
|  | |  | 905.532 | 22 |  |  |
|  | |  | 579.336 | 22 |  |  |
| KaAAPATGGVKpKaPHR | | 519.969 | 1245.706 | 22 | 4.35 |  |
|  | |  | 919.547 | 22 |  |  |
|  | |  | 579.336 | 22 |  |  |
| KaAAPATGGVKaKpPHR | | 519.971 | 1245.706 | 22 | 4.35 |  |
|  | |  | 919.547 | 22 |  |  |
|  | |  | 593.352 | 22 |  |  |
| KaAAPATGGVKaKaPHR | | 515.295 | 1231.691 | 22 | 3.80 |  |
|  | |  | 905.532 | 22 |  |  |
|  | |  | 579.336 | 22 |  |  |
| VTIQPKpDMQLAR | | 485.938 | 1014.540 | 21 | 8.85 |  |
|  | |  | 917.487 | 21 |  |  |
|  | |  | 733.366 | 21 |  |  |
|  | |  | 618.339 | 21 |  |  |
|  | |  |  |  |  |  |
| VTIQPKaDMQLAR | | 481.266 | 1000.524 | 21 | 8.75 |  |
|  | |  | 903.472 | 21 |  |  |
|  | |  | 733.366 | 21 |  |  |
|  | |  | 618.339 | 21 |  |  |
| **H3 G34R mutant** | |  |  |  |  |  |
| KpAAPATGR | | 414.240 | 643.352 | 15 | 0.60 |  |
|  | |  | 572.315 | 15 |  |  |
|  | |  | 501.278 | 15 |  |  |
| KaAAPATGR | | 407.233 | 643.352 | 15 | 0.45 |  |
|  | |  | 572.315 | 15 |  |  |
|  | |  | 501.278 | 15 |  |  |
| VKpKpPHR | | 438.774 | 777.473 | 16 | 0.95 |  |
|  | |  | 593.352 | 16 |  |  |
|  | |  | 284.197 | 16 |  |  |
| VKpKaPHR | | 431.767 | 763.457 | 16 | 0.70 |  |
|  | |  | 579.336 | 16 |  |  |
|  | |  | 284.197 | 16 |  |  |
| VKaKpPHR | | 431.769 | 763.457 | 16 | 0.70 |  |
|  | |  | 593.352 | 16 |  |  |
|  | |  | 270.181 | 16 |  |  |
| VKaKaPHR | | 424.759 | 749.442 | 16 | 0.40 |  |
|  | |  | 579.336 | 16 |  |  |
|  | |  | 270.181 | 16 |  |  |

**H3 G34V mutant**

| KpAAPATGVVKpKpPHR | 543.326 | 1301.769 | 23 | 7.12 |  |
| --- | --- | --- | --- | --- | --- |
|  |  | 975.610 | 23 |  |  |
|  |  | 593.352 | 23 |  |  |
| KpAAPATGVVKpKaPHR | 538.652 | 1287.753 | 23 | 6.60 |  |
|  |  | 961.594 | 23 |  |  |
|  |  | 579.336 | 23 |  |  |
| KpAAPATGVVKaKpPHR | 538.654 | 1287.753 | 23 | 6.60 |  |
|  |  | 961.594 | 23 |  |  |
|  |  | 593.352 | 23 |  |  |
| KaAAPATGVVKpKpPHR | 538.656 | 1301.769 | 23 | 6.60 |  |
|  |  | 975.610 | 23 |  |  |
|  |  | 593.352 | 23 |  |  |
| KpAAPATGVVKaKaPHR | 533.980 | 1273.738 | 23 | 6.15 |  |
|  |  | 947.579 | 23 |  |  |
|  |  | 579.336 | 23 |  |  |
| KaAAPATGVVKaKpPHR | 533.982 | 1287.753 | 23 | 6.15 |  |
|  |  | 961.594 | 23 |  |  |
|  |  | 593.352 | 23 |  |  |
| KaAAPATGVVKpKaPHR | 533.984 | 1287.753 | 23 | 6.15 |  |
|  |  | 961.594 | 23 |  |  |
|  |  | 579.336 | 23 |  |  |
| KaAAPATGVVKaKaPHR | 529.311 | 1273.738 | 22 | 5.50 |  |
|  |  | 947.579 | 22 |  |  |
|  |  | 579.336 | 22 |  |  |

*p= propionylated, a= acetylated
