# Supplementary material for: Surprising phenotypic diversity of cancer-associated mutations of Gly 34 in the histone H3 tail
Source: eLife. 2021 Feb 1;10:e65369. doi: 10.7554/eLife.65369 (PMC7872514; doi:10.7554/eLife.65369)
Supplement: Transparent reporting form [file elife-65369-transrepform.docx]

***eLife’s* transparent reporting form**

We encourage authors to provide detailed information *within their submission* to facilitate the interpretation and replication of experiments. Authors can upload supporting documentation to indicate the use of appropriate reporting guidelines for health-related research (see [EQUATOR Network](http://www.equator-network.org/%20)), life science research (see the [BioSharing Information Resource](https://biosharing.org/)), or the [ARRIVE guidelines](http://www.plosbiology.org/article/info:doi/10.1371/journal.pbio.1000412) for reporting work involving animal research. Where applicable, authors should refer to any relevant reporting standards documents in this form.

If you have any questions, please consult our Journal Policies and/or contact us: [editorial@elifesciences.org](mailto:editorial@elifesciences.org).

**Sample-size estimation**

- You should state whether an appropriate sample size was computed when the study was being designed
- You should state the statistical method of sample size computation and any required assumptions
- If no explicit power analysis was used, you should describe how you decided what sample (replicate) size (number) to use

Please outline where this information can be found within the submission (e.g., sections or figure legends), or explain why this information doesn’t apply to your submission:

Sample sizes were not computed during study design. Sample sizes were largely determined by prior experience, and are documented either in main text or in fig legends.

RNA-seq analysis (Fig 4 and 6) –triplicate biological replicates previously yielded sufficient sample size for statistical assessment of data (Yadav et al 2017).

Histone purifications for mass spec (Fig. 1) used 6-9 biological replicates (Fig 6) used 4 biological replicates.

K36 methylation westerns used 2-8 biological replicates per sample.

ChIPs used 6 biological replicates based on prior experience.

Homologous recombination assays (Fig. 6) used 3 biological replicates to obtain statistically relevant data.

Chromosome segregation analyses used 4-8 biological replicates per genotype (Fig 2 and 3) , 3 biological replicates (Fig 6).

**Replicates**

- You should report how often each experiment was performed
- You should include a definition of biological versus technical replication
- The data obtained should be provided and sufficient information should be provided to indicate the number of independent biological and/or technical replicates
- If you encountered any outliers, you should describe how these were handled
- Criteria for exclusion/inclusion of data should be clearly stated
- High-throughput sequence data should be uploaded before submission, with a private link for reviewers provided (these are available from both GEO and ArrayExpress)

Please outline where this information can be found within the submission (e.g., sections or figure legends), or explain why this information doesn’t apply to your submission:

No. of biological or technical replicates is clearly stated within the figure legends for each experiment. The only data that was excluded was for mass spec analysis of acetylation of H3K27 (Fig 1D and in vitro Fig 1F) as it was highly variable between experiments and biological replicates. RNAseq analysis is included in GEO submission # GSE162572

**Statistical reporting**

- Statistical analysis methods should be described and justified
- Raw data should be presented in figures whenever informative to do so (typically when N per group is less than 10)
- For each experiment, you should identify the statistical tests used, exact values of N, definitions of center, methods of multiple test correction, and dispersion and precision measures (e.g., mean, median, SD, SEM, confidence intervals; and, for the major substantive results, a measure of effect size (e.g., Pearson's r, Cohen's d)
- Report exact p-values wherever possible alongside the summary statistics and 95% confidence intervals. These should be reported for all key questions and not only when the p-value is less than 0.05.

Please outline where this information can be found within the submission (e.g., sections or figure legends), or explain why this information doesn’t apply to your submission:

Statistical analyses used are listed in methods. Page 24, line 30-32.

(For large datasets, or papers with a very large number of statistical tests, you may upload a single table file with tests, Ns, etc., with reference to sections in the manuscript.)

**Group allocation**

- Indicate how samples were allocated into experimental groups (in the case of clinical studies, please specify allocation to treatment method); if randomization was used, please also state if restricted randomization was applied
- Indicate if masking was used during group allocation, data collection and/or data analysis

Please outline where this information can be found within the submission (e.g., sections or figure legends), or explain why this information doesn’t apply to your submission:

Chromosome stability assays were performed with masking of strain identities (Page 20), as were HR assays and gamma irradiation plating experiments (Page 24).

**Additional data files (“source data”)**

- We encourage you to upload relevant additional data files, such as numerical data that are represented as a graph in a figure, or as a summary table
- Where provided, these should be in the most useful format, and they can be uploaded as “Source data” files linked to a main figure or table
- Include model definition files including the full list of parameters used
- Include code used for data analysis (e.g., R, MatLab)
- Avoid stating that data files are “available upon request”

Please indicate the figures or tables for which source data files have been provided:

RNAseq source data (Fig 4 and Fig 6) is provided in the GEO dataset.
